# Interactions Via α_2_β_1_ Cell Integrin May Protect against the Progression of Airway Structural Changes in Asthma

**DOI:** 10.3390/ijms22126315

**Published:** 2021-06-12

**Authors:** Stanislawa Bazan-Socha, Bogdan Jakiela, Joanna Zuk, Jacek Zarychta, Jerzy Soja, Krzysztof Okon, Sylwia Dziedzina, Lech Zareba, Jerzy Dropinski, Krzysztof Wojcik, Agnieszka Padjas, Cezary Marcinkiewicz, Jan G. Bazan

**Affiliations:** 1 Faculty of Medicine, Department of Internal Medicine, Jagiellonian University Medical College, 31-066 Krakow, Poland; b.jakiela@uj.edu.pl (B.J.); j.zuk@uj.edu.pl (J.Z.); jzar@mp.pl (J.Z.); jerzysoja1@gmail.com (J.S.); sylwiaz49@poczta.fm (S.D.); ddropinski@gmail.com (J.D.); krzysztof.wojcik@uj.edu.pl (K.W.); agnieszkapadjas@gmail.com (A.P.); 2 Pulmonary Hospital, 34-500 Zakopane, Poland; 3 Faculty of Medicine, Department of Pathology, Jagiellonian University Medical College, 31-531 Krakow, Poland; k.okon@uj.edu.pl; 4 College of Natural Sciences, Institute of Computer Science, University of Rzeszów, 35-310 Rzeszów, Poland; lzareba@ur.edu.pl (L.Z.); bazan@ur.edu.pl (J.G.B.); 5 Department of Bioengineering, College of Engineering, Temple University, Philadelphia, PA 19122, USA; cmarcink@temple.edu

**Keywords:** asthma, airway remodeling, computed tomography, biomarkers, histology

## Abstract

Increased airway wall thickness and remodeling of bronchial mucosa are characteristic of asthma and may arise from altered integrin signaling on airway cells. Here, we analyzed the expression of β_1_-subfamily integrins on blood and airway cells (flow cytometry), inflammatory biomarkers in serum and bronchoalveolar lavage, reticular basement membrane (RBM) thickness and collagen deposits in the mucosa (histology), and airway geometry (CT-imaging) in 92 asthma patients (persistent airflow limitation subtype: *n* = 47) and 36 controls. Persistent airflow limitation was associated with type-2 inflammation, elevated soluble α_2_ integrin chain, and changes in the bronchial wall geometry. Both subtypes of asthma showed thicker RBM than control, but collagen deposition and epithelial α_1_ and α_2_ integrins staining were similar. Type-I collagen accumulation and RBM thickness were inversely related to the epithelial expression of the α_2_ integrin chain. Expression of α_2_β_1_ integrin on T-cells and eosinophils was not altered in asthma. Collagen I deposits were, however, more abundant in patients with lower α_2_β_1_ integrin on blood and airway CD8^+^ T-cells. Thicker airway walls in CT were associated with lower α_2_ integrin chain on blood CD4^+^ T-cells and airway eosinophils. Our data suggest that α_2_β_1_ integrin on inflammatory and epithelial cells may protect against airway remodeling advancement in asthma.

## 1. Introduction

Airway remodeling refers to structural and functional changes in bronchial walls caused by inflammation and repeated cycles of injury and repair [1]. In asthma, it is characterized by structural and functional alterations of airway epithelium and subepithelial fibrosis, with thickening of the basement membrane and increased deposition of extracellular matrix (ECM) proteins in submucosa being the most prominent features [2]. Thickening of the basement membrane occurs mainly in the lamina reticularis layer named reticular basement membrane (RBM), which is composed of collagen fibers (mostly type III) produced primarily by underlying connective tissue cells [2,3]. There are more than twenty different subtypes of collagen, of which collagens I and III constitute the structural framework of lungs. In the asthmatic airways, collagen deposits accumulate in the basement membrane in a disorganized and fragmented form [4]. In contrast, type IV collagen is present mainly in the basal lamina, where it forms the central platform for anchoring epithelial cells [5].

Increased smooth muscle mass and increased airway wall stiffness is another consistent trait of airway remodeling that correlates with impaired lung function in asthma [6,7]. However, it may also protect against exaggerated responses to allergens and other inflammatory stimuli, preventing immediate bronchoconstriction [8]. Progression of remodeling has been linked with chronic airway inflammation, although the causal relationship is uncertain [9]. Nevertheless, repeated mechanical stress may cause bronchial wall structural changes even in the absence of inflammation [10].

Various features of airway remodeling, such as loss of epithelial cells and mucus cell hyperplasia, RBM thickening, and smooth muscle hypertrophy, can be described using a histological examination of airway mucosa [11]. However, emerging non-invasive methods, including lung computed tomography (CT) imaging, provide important remodeling measures that might be useful in quantifying bronchial wall thickening in a standardized and more comprehensive way [12,13].

Integrins are a large family of transmembrane glycoproteins that mediate cell–cell and cell–ECM interactions, and thus are involved in a broad range of cellular processes, including cell adhesion, migration, and proliferation [14]. Each integrin subfamily is characterized by a common β subunit and non-covalently associated variable α chain. The β_1_-subfamily includes, among others, integrins α_1_β_1_, α_2_β_1_, α_10_β_1_, and α_11_β_1_ that are characterized by high-affinity binding to GFOGER-like motifs in collagen. Integrins α_1_β_1_ and α_2_β_1_, which show a preference for binding to type IV and I collages, respectively, are essential members within that subset [14]. They are expressed on various cells, including vascular and epithelial cells, fibroblasts, and activated T-cells, responsible for the cell interactions with collagen fibers in basement membranes and ECM [15,16]. Therefore, both may be involved in asthma airway remodeling pathology. In an early report, Schuliga et al. [17] showed that interaction of airway smooth muscle cells with type I collagen via α_2_β_1_ integrin potentiated conversion of plasminogen into plasmin with subsequent degradation of ECM proteins. Furthermore, it has been demonstrated that in vitro inhibition of α_2_β_1_ integrin on fibroblasts enhanced their proliferation together with excessive lung ECM deposition and tissue fibrosis [18], supporting the concept of the potential antifibrotic role of α_2_β_1_ integrin in airways.

A growing body of evidence suggests that T-cell interactions with ECM proteins in perivascular tissues are essential for regulating the inflammatory response [19]. Furthermore, collagen-binding integrins not involved in cell migration occurred to be crucial costimulatory molecules of effector T cells [19,20]. Moreover, it has been demonstrated that α_2_β_1_ integrin may promote the survival of effector T cells by inhibiting Fas-induced apoptosis [21]. Although naïve T cells express very low levels of α_1_β_1_ and α_2_β_1_ integrins, they become abundant upon in vitro activation [19]. Asthma is an airway disease with a locally limited inflammatory response. However, increased blood levels of inflammatory cytokines and signs of coagulation pathway activation suggest accompanying low-grade systemic inflammation [22,23]. Thus, activated immune and effector cells, such as T-cells and eosinophils, although essential for airway site, may also be found in the systemic circulation likely primed in airways or by circulating inflammatory cytokines.

Given the potential contribution of α_1_β_1_ and α_2_β_1_ collagen-binding integrins in the regulation of ECM environment [24], we have decided to evaluate their role in the airway structural changes in asthma. We investigated their expression on circulating and bronchoalveolar lavage fluid (BAL) inflammatory cells and in bronchial wall biopsies. We also analyzed their relationships with asthma blood and BAL biomarkers, lung function abnormalities, and renowned indices of airway remodeling, including RBM thickness and accumulation of collagen deposits in bronchial mucosa specimens, as well as bronchial wall geometry alterations in CT imaging. According to our knowledge, such studies have not been performed to date.

## 2. Results

### 2.1. Clinical Characteristics and Airway Inflammatory Signature in Asthma Patients with Persistent Airflow Limitation

We analyzed 92 adult, non-smoking asthma patients and 36 controls. Among asthmatics, 47 subjects were characterized by persistent airflow limitation, while 45 had normal spirometry before or after a bronchodilator (non-persistent airflow limitation subtype). All three analyzed groups were similar in demographic variables, including body mass index (BMI), and past smoking, although asthma patients with persistent airflow limitation were older than the remaining asthmatics (Table 1). The studied asthma subtypes did not differ in disease duration and severity, staged according to the Global Initiative for Asthma (GINA) guidelines [25]. Atopy was more frequent in asthmatics. Other comorbidities were equally prevalent in all three analyzed groups, except for gastroesophageal reflux disease (GERD), which was more prevalent in the control individuals (Table 1). 

Asthma patients showed higher serum IgE and increased red blood cell, lymphocyte, and monocyte counts compared to controls (Table 2). In turn, white blood cells and neutrophils were elevated in asthma patients with persistent airflow limitation than in other groups. Furthermore, asthma patients with persistent airflow limitation were characterized by elevated type-2 (T2) inflammatory biomarkers, such as blood and BAL eosinophilia, and serum periostin when comparing the remaining asthmatics (Table 2). Serum levels of a disintegrin and metalloproteinase domain-containing protein (ADAM)-33 were also increased in the asthma subtype with persistent airflow limitation (Table 2). 

Other analyzed laboratory variables, not crucial for the primary outcomes, are provided in the Supplementary file (Table S1).

### 2.2. Asthma Is Characterized by Decreased Expression of α_4_ and β_1_ on Circulating Inflammatory Cells and Increased Expression of α_1_ Integrin Chain

**Figure 1:**
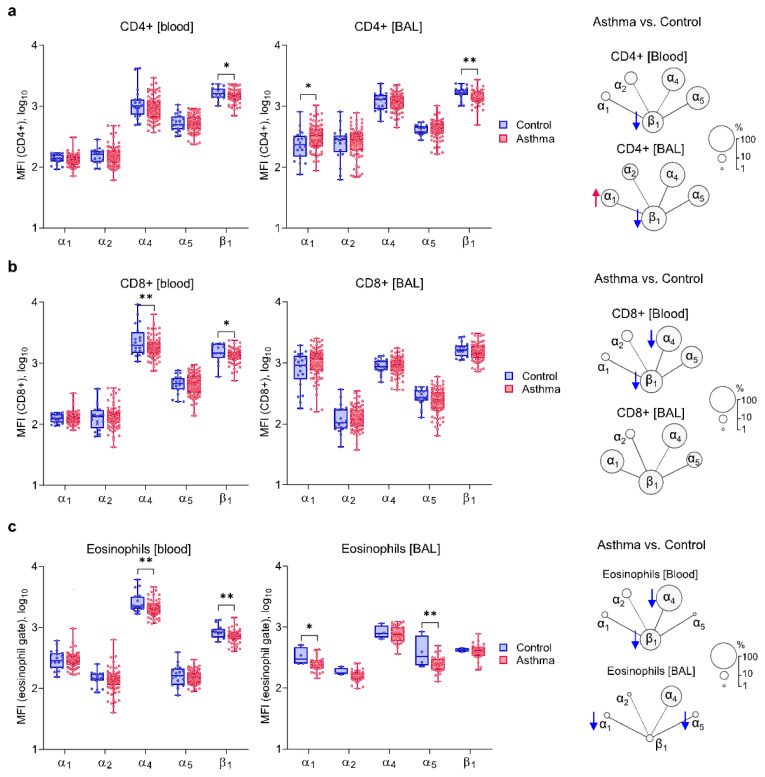
Surface expression of integrin chains on T-cells and eosinophils in asthma and control subjects. Median fluorescent intensities (MFI) of the studied integrin chains in different compartments (on the left) and diagram summarizing the differences in the expression between the two analyzed groups (on the right, circle areas reflect the percentage of cells positive for a given integrin). (**a**,**b**) Asthma patients showed higher expression of α_1_ integrin chain on bronchoalveolar lavage (BAL) CD4^+^ T-cells (red arrow) and decreased expression of β_1_ (blue arrow) on CD4^+^ blood and BAL and CD8^+^ blood T-cells and lower expression of α_4_ on blood CD4^+^ T cells. (**c**) Lower expression of surface α_4_ and β_1_ integrin chains on blood eosinophils and α_1_ and α_5_ on BAL eosinophils in asthma. Data presented as medians and range (T-cells: blood *n* = 108, BAL *n* = 101; eosinophils: blood *n* = 103, BAL *n* = 35; β_1_ was measured in 78% of samples). Statistics: ANCOVA with adjustment for age, sex, and BMI: * *p* < 0.05, ** *p* < 0.01.

First, we analyzed the expression of β_1_-subfamily integrins on blood and BAL inflammatory cells in asthma and control individuals. Surprisingly, blood CD8^+^ T-cells and eosinophils in asthma were characterized by lower expression of α_4_ and β_1_ integrin chains (Figure 1). Furthermore, circulating and airway CD4^+^ T-cells showed lower expression of β_1_ integrin chains, whereas BAL CD4^+^ T-cells additionally had increased expression of α_1_ (Figure 1a, Supplementary Table S2). The α_1_ integrin chain was also present on a higher percentage of blood eosinophils and blood CD8^+^ T-cells (Supplementary Tables S2 and S3).

### 2.3. Similar Expressions of α_1_ and α_2_ Integrin Chains on Blood and BAL Inflammatory Cells of Both Asthma Subsets

**Figure 2:**
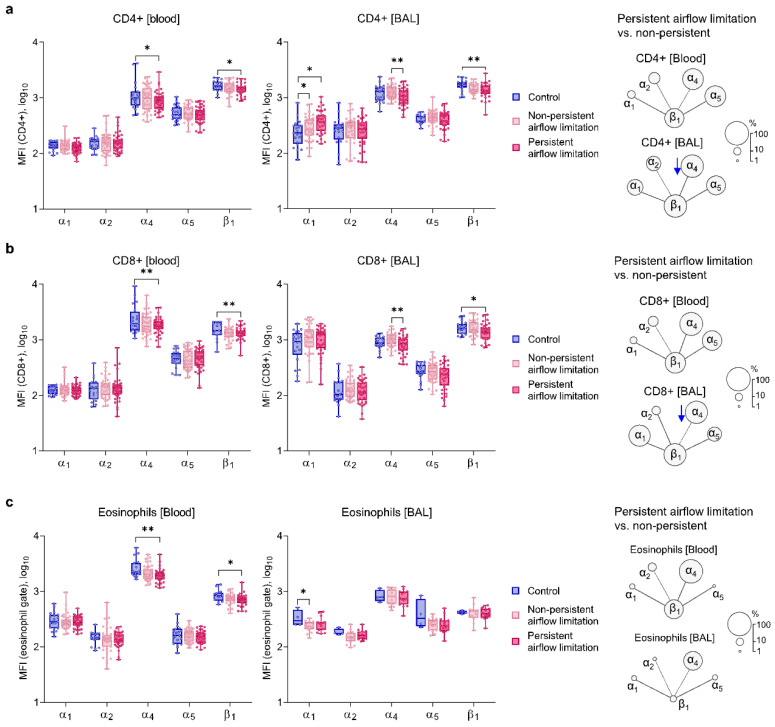
Surface expression (median fluorescence intensity [MFI]) of integrin chains on T-cells and eosinophils in asthma patients with persistent vs. non-persistent airflow limitation and controls. (**a**,**b**) Patients with persistent airflow limitation showed lower expression of α_4_ and β_1_ on both blood T-cell subsets, decreased β_1_ on both BAL T-cell subsets, and increased α_1_ on BAL CD4^+^ T-cells but only compared to controls. They also had lower α_4_ on both BAL T-cell subsets comparing the non-persistent airflow limitation group (blue arrows in the diagram on the right). (**c**) Blood eosinophils of persistent airflow limitation patients had lower α_4_ and β_1_ than controls and no difference compared to the non-persistent airflow limitation subgroup. Data are presented as medians and range (T-cells: blood *n* = 108, BAL *n* = 101; eosinophils: blood *n* = 103, BAL *n* = 35; β_1_ was measured in 78% of samples). Statistics: ANCOVA with adjustment for age, sex, and BMI: * *p* < 0.05, ** *p* < 0.01.

Next, we compared the expression of studied integrin subunits in persistent vs. non-persistent airflow limitation patients and controls. In comparison to the control group, patients with persistent airflow limitation showed lower expression of α_4_ and β_1_ on both blood T-cell subsets and eosinophils and decreased β_1_ on BAL T-cells. Additionally, they were characterized by increased expression of α_1_ integrin chain on BAL CD4^+^ T cells, albeit once again only compared to the controls (Figure 2, Supplementary Table S2). They also had lower α_4_ on BAL T-cells than those with non-persistent airflow limitation (Figure 2a,b). Compared to controls, the latter group had elevated α_1_ on BAL CD4^+^ T cells (Figure 2a). 

Blood eosinophils in patients with persistent airflow limitation also had a lower expression of α_4_ and β_1_ than in controls; they also did not differ from the non-persistent airflow limitation subgroup (Figure 2c).

### 2.4. Increased Systemic Levels of Soluble α_2_ Chain in Asthma Patients with Persistent Airflow Limitation

Some immune receptors can be detached from the cell surface and enter the systemic circulation. Here, we measured systemic levels of α_1_ and α_2_ integrin chains to investigate whether their amounts reflect inflammation process activity and advancement of remodeling in asthma. The serum levels of the studied integrin subunits were similar in the studied groups, except for the moderate increase in α_2_ concentration (by ~45%) in asthma patients with persistent airflow limitation vs. the remaining asthmatics (Table 2). Interestingly, in asthma patients, α_2_ was weakly inversely associated with a forced expiratory volume in 1 s (FEV_1_)/vital capacity (VC) index (R = −0.24, *p* = 0.04). Additionally, levels of α_2_ in the serum positively correlated with the surface expression of this integrin on blood and BAL CD8^+^ T-cells (blood: R = 0.38, *p* = 0.02; BAL: R = 0.43, *p* = 0.02) and blood eosinophils (R = 0.3, *p* = 0.02). Interestingly, similar associations were demonstrated between circulating α_1_ levels and α_1_ integrin expression on blood and BAL CD8^+^ T-cells (blood: R = 0.31, *p* = 0.006; BAL: R = 0.24, *p* = 0.03), blood CD4^+^ T cells (R = 0.23, *p* = 0.03), and blood eosinophils (R = 0.3, *p* < 0.001). We also noticed a weak positive correlation between circulating α_2_ integrin chain concentrations and inflammatory blood biomarkers, such as interleukin (IL)-6 (R = 0.26, *p* = 0.02), IL-12p70 (R = 0.32, *p* = 0.003), and ADAM-33 (R = 0.3, *p* = 0.005).

### 2.5. Similar Bronchial Mucosa Expression of α_1_ and α_2_ Integrin Subunits and RBM Thickness in Asthma Patients with Persistent and Non-Persistent Airflow Limitation

**Figure 3:**
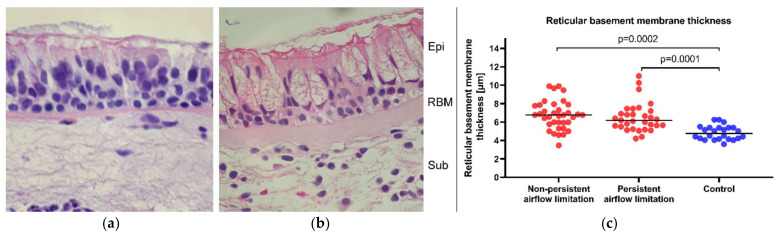
Reticular basement membrane (RBM) thickness measurements. The representative photomicrograph of hematoxylin and eosin-stained bronchial mucosa specimen from (**a**) a control individual and (**b**) an asthma patient—the picture depicts typical asthma remodeling changes, including mucus cell hyperplasia and RBM thickening; abbreviations: Epi—epithelial cells, RBM—reticular basement membrane, Sub—submucosa; (**c**) RBM measurements in all three analyzed groups; *n* = 35 non-persistent airflow limitation patients, *n* = 36 persistent airway obstruction patients, and *n* = 24 control. Statistics: one-way covariance analysis (ANCOVA) adjusted for age, sex, and body mass index.

To find out if persistent airflow limitation associates with the structural remodeling alterations of the bronchial mucosa and local expression of α_1_ and α_2_ integrins, we assessed RBM thickness, collagen deposits, and reactivity of α_1_ and α_2_ integrin subunits on airway epithelium in bronchial biopsy specimens. Interestingly, the thickness of the RBM layer was increased in both asthma subsets compared to the control group (Figure 3) and did not depend on spirometry values (e.g., for FEV_1_/VC: R = −0.13, *p* = 0.56). In turn, as the deposition of type I and IV collagen was similar in asthma patients and control subjects, so too was the expression of α_1_ and α_2_ on bronchial epithelial cells (Table 3). Similarly, the expression of the studied collagen types and integrin subunits did not depend on lung function tests (e.g., for FEV_1_/VC: *p* = 0.2 and *p* = 0.96, for collagen I and IV, respectively; and *p* = 0.68 and *p* = 0.52 for α_1_ and α_2_ integrin chain staining on airway epithelial cells, respectively; statistics: one-way covariance analysis [ANCOVA] with adjustment for age, sex, and BMI). 

Surprisingly, RBM thickness was not associated with clinical parameters of asthma, such as disease duration or severity. It was, however, predicted by the concentration of BAL periostin (β = 0.5 [95% CI: 0.28–0.7], after adjustment for potential confounders). Similarly, collagen I accumulation was not related to asthma duration and severity but patients with stronger collagen I staining (scored 2 or 3 in immunohistochemistry [64% of asthma subjects]) had lower FEV_1_ after a bronchodilator (1.75 [1.97–3.34] vs. 2.23 [1.72–2.81], *p* = 0.03). Additionally, increased collagen I accumulation was related to the higher percentage of eosinophils in BAL (β = 0.22 [95% CI: 0.11–0.34]) also confirmed by comparison of subgroups with high vs. low collagen I staining (1 [0.1–2] vs. 0.5 [0.1–1.5] %, *p* = 0.03, respectively). 

Interestingly, higher collagen I in bronchial mucosa was related to the lower α_2_ integrin staining on bronchial epithelial cells (*p* = 0.02, χ^2^ test). Moreover, asthma patients with low α_2_ immunoreactivity on epithelial cells (scores 0 or 1 [67% of asthma subjects]) compared to those with stronger staining (score 2 or 3) showed thicker RBM (6.79 [5.82–7.94] vs. 5.64 [5.17–6.66] μm, *p* = 0.02) and increased concentration of IL-6 in BAL (0.78 [0.17–1.39] vs. 0.51 [0.01–0.89] pg/mL, *p* = 0.02). 

We also searched for potential linkages between signs of remodeling in bronchial mucosa histology and integrin subunits expression on circulating and BAL inflammatory cells. As it turned out, RBM thickness was not related to the expression of the studied integrins on blood or BAL inflammatory cells. In turn, the percentage of stroma area showing collagen I reactivity tended to be inversely associated with α_2_ on BAL CD8^+^ T cells (R = −0.33, *p* = 0.004). Furthermore, patients with stronger collagen I staining (scored 2 or 3) were characterized by a lower expression of α_2_ on blood CD8^+^ T-cells (median 1.2-fold decrease in MFI, *p* = 0.007).

Interestingly, a higher abundance of type IV collagen deposits (patients with staining scores 2 or 3 [43% of asthma subjects]) was associated with lower α_4_ subunit expression on blood (but not BAL) CD4^+^ and CD8^+^ T-cells (median 1.3-fold decrease in MFI for both, *p* = 0.007 and *p* = 0.02, respectively). 

We did not document any significant association between the expression of α_1_ or α_2_ integrins on airway epithelial cells and β_1_-subfamily integrin chains on airway or blood inflammatory cells. 

### 2.6. Persistent Airflow Limitation in Asthma Is Linked with Computed Tomography Parameters of the Bronchial Tree, Suggesting More Advanced Airway Wall Remodeling

**Figure 4:**
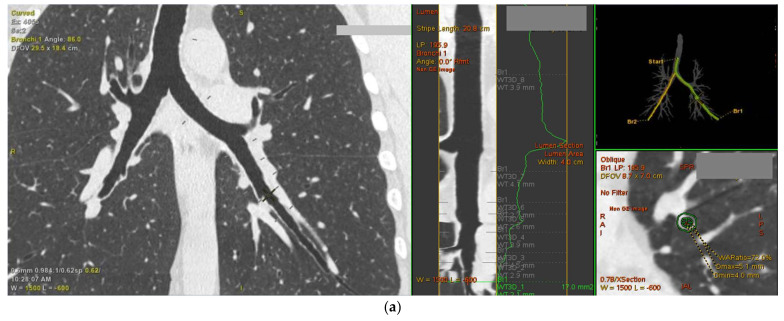

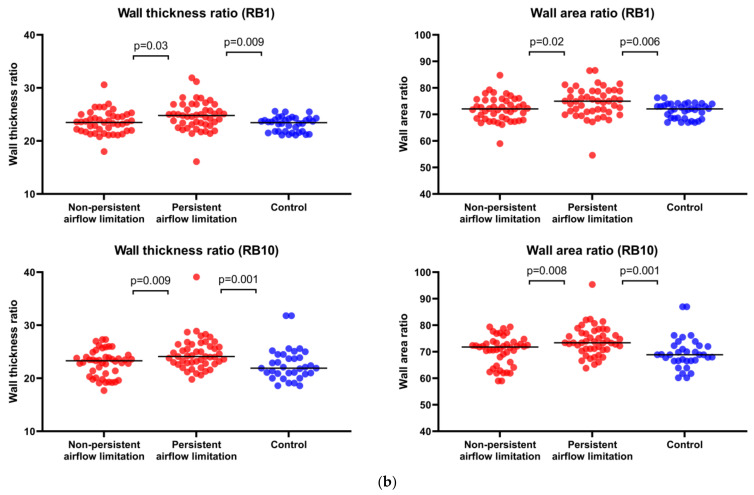
Computed tomography airway geometry parameters in asthma patients with persistent and non-persistent airflow limitation, and controls. (**a**) Automatic evaluation of airway geometry parameters in lung computed tomography imaging. After identifying the studied bronchus, the operator placed a seed point in the airway lumen (left side), while the software automatically quantified airway geometry parameters, including lumen and airway diameters, wall thickness, lumen and wall area (bottom right side), and calculated wall thickness and wall area ratios. (**b**) Wall thickness ratio (WTR) and wall area ratio (WAR) of the right upper lobe apical segmental bronchus (RB1) (top) and the right lower lobe basal posterior bronchus (RB10) (bottom); *n* = 45 non-persistent airflow limitation patients, *n* = 47 persistent airway obstruction patients, and *n* = 36 control. Statistics: one-way covariance analysis (ANCOVA) adjusted for age, sex, and body mass index.

In the final part of the study, we investigated the potential linkage between various CT imaging parameters referring to the altered bronchial geometry and histological traits of airway remodeling, expression of α_1_ and α_2_ integrins on airway epithelium, and on BAL or blood inflammatory cells. Airway geometry parameters were automatically measured in lung CT scans, which allowed for standardized calculation of wall thickness ratios (WTR) and wall area ratios (WAR) at designated regions of the bronchial tree (representative image shown in Figure 4a).

As expected, asthma patients with persistent airflow limitation were characterized by significantly higher WTR and WAR in RB1 and RB10 compared to other studied groups (Figure 4b). Furthermore, they also had thicker bronchial walls in the LB1 + 2 region and altered airway geometry in the LB10 bronchus, which corresponded to a lower lumen diameter and area resulting in increased WAR and WTR, albeit only in comparison to the controls (Table 4). 

Surprisingly, airway imaging parameters did not correlate well with the histological traits of airway remodeling. In asthma patients, we demonstrated only a weak correlation between RBM thickness and bronchial wall thickness in RB10 (R = 0.3, *p* = 0.04; β = 0.27 [95% CI: 0.15–0.4], after adjustment for potential confounders). Moreover, the percentage of stroma area showing reactivity for collagen I was inversely associated with WAR and WTR of RB10 (R = −0.4, *p* = 0.001, for both), suggesting that collagen I abundance in the small airways might be related to more rigid and compressed their structure, thus thinner walls in CT imaging.

Airway thickness parameters in the RB1 region, such as WAR, correlated weakly positively with the systemic levels of IL-10 (R = 0.31, *p* = 0.007) and IL-17A (R = 0.3, *p* = 0.008), but also with the serum concentration of ADAM-33 (R = 0.26, *p* = 0.02). Furthermore, they were inversely associated with the expression of α_2_ on blood CD4^+^ T-cells (e.g., for WAR: R = −0.25, *p* = 0.03) and BAL eosinophils (e.g., for WAR: R = −0.42, *p* = 0.02), suggesting, once again, a potential linkage between lower α_2_ on inflammatory cells and progression to airway remodeling.

## 3. Discussion

In the present study, we analyzed airway remodeling in asthma using CT imaging of large and peripheral bronchi and histological examination of the bronchial mucosa. We aimed to identify structural and cellular factors associated with persistent airflow limitation and investigated the potential linkage between the advancement of airway remodeling and the expression of integrin subunits on epithelial and inflammatory cells. Asthma patients with persistent airflow limitation were characterized by altered airway geometry, mainly increased WTR and WAR in the right bronchi, compared to patients without persistent airflow limitation, which stays in line with previously published reports [12,13,27]. In contrast, well-defined signs of remodeling in bronchial mucosae, such as RBM thickness and collagen deposits [11], were comparable in both studied subgroups. 

Our data suggest that RBM thickening likely progresses independently of the decline in lung function and changes in airway geometry. For example, the thickness of the RBM layer was not associated with spirometry values or renowned CT-imaging measures of airway remodeling, such as WTR and WAR. That implies that RBM changes in asthma have possibly only a weak impact on the clinical course of the disease. Still, the biological role of the thicker RBM in asthma remains primarily uncovered. This phenomenon has been suggested to serve as an additional barrier, equalizing increased epithelial leakiness and fragility [28]. However, Rijt et al. [29] demonstrated in the murine model of chronic airway inflammation that a thicker basement membrane might promote dendritic cell activation and enhance further sensitization. Therefore, novel therapies aimed at stabilizing RBM to some extent, such as biological therapy or kinase inhibitors, might be a therapeutic option for particularly severe asthma individuals [30]. However, such an approach needs comprehensive elucidation of the role of thicker RBM in asthma, its determinants, and dynamics in progression. In our study, the thickening of the RBM did not depend on the asthma duration, suggesting that the RBM alteration occurs early in the disease, likely as a compensatory response to ongoing but yet not recognized airway inflammation. This observation stays in line with early research conducted by Payne et al. [31], who showed that RBM thickening could occur in children with a median age of 13 years or even in preschool-age children with recurring wheezing episodes [32]. 

Asthma patients with persistent airflow limitation showed higher airway and systemic biomarkers of T2 inflammation (e.g., blood and BAL eosinophilia and circulating periostin), increased blood neutrophil counts, and serum concentration of ADAM-33. In contrast, thicker walls in CT correlated with increased circulating inflammatory biomarkers, such as IL-10, IL-17A, and ADAM-33, but not with the specific T2 biomarkers. On the other hand, airway T2 markers were associated with the remodeling at the level of the bronchial mucosa. For example, BAL periostin occurred to be a strong predictor of thicker RBM, whereas BAL eosinophilia was associated with higher collagen I accumulation. One reason for this relationship may be that airway epithelial cells are the primary source of periostin, which may, in turn, stimulate TGF-β release and activation of lung fibroblasts to produce ECM proteins [28]. On the contrary, the biological role of ADAM-33 in asthma pathology remains unknown. It has been reported to be overexpressed in the airway epithelium, fibroblasts, and smooth muscle cells. In the airways, it promotes angiogenesis, cell proliferation, and airway structural changes, which are reflected by the thicker bronchial walls and lower spirometry values, which stay in line with our results [33]. These data suggest altogether that separate mechanisms may operate in airway remodeling pathology, depending on whether they act on the airway wall structure (e.g., smooth muscle hypertrophy) or at the site of bronchial mucosa (e.g., RBM thickness and collagen deposition).

In search for potential regulation of airway remodeling, we analyzed the expression of integrin β_1_-subfamily components both on the airway epithelium and infiltrating cells. First, we demonstrated distinct changes in their expression on airway inflammatory cells in asthma, e.g., upregulated α_1_ subunit on BAL CD4^+^ T-cells, which suggests their activation and potential priming for binding to ECM components. In turn, peripheral blood cells did not differ in collagen-binding integrins among study groups. That is contrary to our previous reports, demonstrating their potential upregulation on circulating inflammatory cells in asthma [34,35]. The discrepancy is likely related to the control group selection. It includes non-asthma subjects referred for bronchoscopy due to the differential respiratory tract symptoms, particularly cough, whereas the previous research represented healthy individuals only. Additionally, here, we performed staining in the whole blood to prevent in vitro cell activation, which likely occurred during immunomagnetic cell separation in our earlier report [35]. However, the most striking results were obtained when we analyzed airway epithelial expression of collagen-binding integrins related to histological traits of airway remodeling. Surprisingly, RBM thickness and collagen I accumulation in the bronchial mucosa were inversely associated with the expression of α_2_ on airway epithelial cells. Furthermore, collagen I deposits were negatively related to the expression of α_2_ on blood and BAL CD8^+^ T-cells, while lung CT airway remodeling indices correlated inversely with the expression of α_2_ integrin chain on blood CD4^+^ T-cells and BAL eosinophils. Taken together, our data suggest that collagen I interaction via α_2_β_1_ integrin on airway epithelial and infiltrating T-cells may play a protective role against the deposition of ECM components. Interestingly, a similar regulatory mechanism of integrin-ECM interaction has been previously shown for airway smooth muscle cells, which participated more efficiently in ECM degradation after collagen I binding to α_2_β_1_ integrin [17]. 

Conversely, increased circulating α_2_ integrin chain in patients with persistent airflow limitation suggests a putative mechanism promoting the advancement of airway remodeling. We do not know how to explain that association; however, one explanation may be that soluble α_2_β_1_ integrin might penetrate inflammatory airways, binding to the extracellular matrix components and saturating its natural ligand, i.e., collagen I. Thus, it may act as an inhibitor of α_2_β_1_ integrin localized on infiltrating or mesenchymal cells. However, extensive experimental and observational studies are needed to verify the above hypothesis. 

Interestingly, our results imply that CD8^+^ T-cells should be regarded as potential effectors linked with airway remodeling in asthma, which is a novel finding. It has been well established that CD4^+^ T lymphocytes play a crucial role in the initiation, progression, and persistence of asthma [36]. In turn, the part of CD8^+^ T cells is less understood. However, some animal studies have demonstrated that CD8^+^ T cells may moderate inflammation and suppress airway hypersensitivity via modulation of T2 immune response [36]. Our data suggest that CD8^+^ T-cells may also play a regulatory role in human asthma pathology, e.g., protecting against enhanced deposition of ECM proteins, e.g., via α_2_β_1_ integrin receptor. Nevertheless, the exact mechanism of how CD8^+^ T-cells contribute to that process needs further examination. 

### Limitations of the Study

Patients with persistent airflow limitation were older, although they were similar in asthma severity and duration compared to the non-persistent airflow limitation subgroup. Control subjects constitute non-asthma individuals; however, they had some respiratory tract symptoms, particularly cough. Since bronchoscopy was an essential part of the study, it could not be performed in healthy individuals due to ethical reasons. Therefore, asthma and control comparisons, particularly regarding inflammatory biomarkers, need to be taken with caution. Statistical associations reported here may not necessarily indicate cause–effect relationships. Finally, the clinical relevance of demonstrated alterations in terms of disease severity, progression, and relation to airway remodeling requires further investigation. 

## 4. Materials and Methods

### 4.1. Patients 

We enrolled 120 white, non-smoking patients with clinically stable asthma, aged 18–70 years: 60 with persistent airflow limitation and 60 with normal spirometry before or after a bronchodilator (i.e., non-persistent airflow limitation subtype). Diagnosis of asthma was established according to the GINA guidelines [25]. Persistent airflow limitation was defined as a FEV_1_/VC index below 0.7 or FEV_1_ lower than 0.8 of predicted value after bronchodilator. All asthma medications, except biological treatment, were permitted, including oral corticosteroids at a daily dose equivalent to ≤10 mg of prednisolone, unless the dose was unchanged during the preceding three months. Asthma patients were not exacerbated in the last six months. The severity of asthma was categorized according to the GINA guidelines [25]; definitions are provided in the Supplementary Data.

The control group (*n* = 40) consisted of non-smoking subjects referred for a diagnostic bronchoscope primarily due to the chronic cough, after excluding chronic obstructive pulmonary disease or asthma based on normal spirometry and negative methacholine challenge test. In a few cases, they were diagnosed with hemoptysis, but none of them had bronchial cancer. 

Spirometry and bronchial reversibility test with 400 μg of albuterol were performed in all enrolled subjects according to the American Thoracic Society standards [37], using a Jaeger MasterLab spirometer (Jaeger-Toennies GmbH; Hochberg, Germany). 

Ex-smokers were eligible for the study if they stopped smoking at least five years ago, with a history of fewer than seven pack-years. The exclusion criteria and definitions of comorbidities have been provided in the Supplementary Data. 

The Ethics Committee of the Jagiellonian University approved the study (approval number: KBET/151/B/2013). All subjects gave written informed consent to participate in the study.

### 4.2. Laboratory Investigations

#### 4.2.1. Basic Laboratory Tests

Basic laboratory tests, including complete blood cell and platelet count, fibrinogen, liver enzymes, C-reactive protein, and kidney function, were analyzed by routine laboratory techniques. IgE were measured by latex nephelometry (Siemens, Marburg, Germany). Blood samples were drawn into serum tubes and centrifuged, similarly to BAL at 2000 g for 20 min. Aliquots of serum were stored at −70 °C until analysis.

#### 4.2.2. Asthma and Inflammatory Biomarkers in Peripheral Blood and Bronchoalveolar Lavage Fluid

Commercially available high sensitivity immunoenzymatic assays (ELISAs) were used to measure levels of IL-4, IL-5, IL-6, IL-10, IL-12p70, IL-17A, and interferon (IFN) γ (eBioscience, Inc. San Diego, CA, USA) in serum and BAL. IL-23 (eBioscience) and periostin (Phoenix Pharmaceuticals, Burlingame, CA, USA) were assessed in BAL and serum, while α_1_ and α_2_ integrins (MyBioSource, San Diego, CA, USA, both) and ADAM-33 (Cloud-Clone Corp., Katy, TX, USA) only in serum by standard ELISA kits.

#### 4.2.3. Flow Cytometry of Blood and Bronchoalveolar Lavage Fluid Inflammatory Cells

Blood aliquots (EDTA anticoagulated) were stained with pre-mixed sets of direct monoclonal antibodies detecting surface integrin chains (CD29-APC/Cy7, CD49a-FITC, CD49b-PE, CD49d-PE/Cy7, and CD49e-APC) or appropriate isotype controls, including the same class and subclass of the antibody as that used for a specific integrin chain staining (all from Biolegend, San Diego, CA, USA) gated on CD4^+^ T-cells (CD3-V500, CD4-PerCP, both from BD Biosciences, Franklin Lakes, NJ, USA), CD8^+^ T-cells (CD3-V500, CD8-PerCP; BD Biosciences), or eosinophils (positive for CD45-V450 [leukocyte gate] and CCR3-BV510 [specific marker], negative for CD16-PerCP, all from BD Biosciences). Aliquots of BAL cells were filtered through 40 μm mesh, washed with phosphate buffer, and incubated with similar sets of antibodies. Stained blood and BAL cells were fixed with FACS lysing solution (BD Biosciences), washed with phosphate buffer, and analyzed by multicolor flow cytometer (FACS Canto II, BD Biosciences) using FACS Diva Software. Gating strategy and exemplary blood and BAL staining data are shown in Supplementary Figures S1 and S2, respectively. Data were presented as median fluorescence intensity (MFI) and % of cells showing expression of a studied molecule. Due to technical reasons, flow cytometry measurements were performed in a limited number of patients; details are provided in the Results section in the main manuscript and the Online Supplement. 

### 4.3. Bronchofiberoscopy, Endobronchial Biopsy, and Bronchoalveolar Lavage

Bronchofiberoscopy was performed according to the guidelines of the American Thoracic Society [38] using the bronchofiberoscope BF 1T180 (Olympus, Tokyo, Japan) with local anesthesia (2% lidocaine) and in mild sedation (0.05–0.1 mg fentanyl and 2.5–5 mg midazolam, intravenously). During this procedure, BAL was performed with 200 mL of 0.9% saline given to the right middle lobe bronchus, and 2–3 bronchial biopsy specimens were taken from the right lower lobe (the carina between B9 and B10). Collected tissue samples were immediately fixed in 10% neutral buffered formalin solution (Sigma-Aldrich, Saint Luis, MO, USA) for further histology examination. 

### 4.4. Bronchoalveolar Lavage Fluid Examination

The cytospin preparations (Thermo Scientific, Walthman, MA, USA) were performed from BAL samples and stained with the May-Grunwald Giemsa dye. In each sample, we counted 1000 cells, and the results were presented as percentages of all inflammatory cells (excluding epithelial cells). The BAL samples were centrifuged, inflammatory cells were analyzed in flow cytometry, whereas the supernatant was frozen in aliquots and stored at −70 °C until analysis.

### 4.5. Histologic Examination 

Histological investigations were analyzed blindly by an independent pathologist (K.O.). Tissue specimens were processed routinely and embedded in paraffin. The 2 μm sections were cut and stained by hematoxylin and eosin. The slides were photographed by the Nikon D5300 camera attached to the Zeiss Axioscope microscope (100× oil immersion lens). The images were acquired along the biopsy’s epithelial surface, including the entire basement membrane, and analyzed by the AnalySIS 3.2 software (Soft Imaging System GmbH, Muenster, Germany) with a custom-built application written in Imaging C language. The RBM thickness was measured with an arbitrary distance software tool of the AnalySIS system. For each patient, 30 or more individual RBM measurements were performed at intervals of 9.5 μm. Results were expressed as a harmonic mean. More detail on RBM layer measurements, including a definition of the harmonic mean, is provided in our previous publication [12].

The immunohistochemistry (IHC) was performed from the paraffin blocks also, in 2 μm sections. The results were scored semi-quantitatively. UltraVision Quanto Detection system with HRP DAB (Thermo Fisher Scientific, Waltham, MA, USA) and hematoxylin as counterstaining were applied for color detection. For identifying α_1_ and α_2_ integrins, we used rabbit polyclonal anti-α_1_ and mouse monoclonal anti-α_2_ integrin antibodies (Abcam, Cambridge, United Kingdom, both). We took only epithelial cells staining into account, which was scored as 0 (lack of staining), 1 (very faint staining), 2 (linear reaction along cellular membranes), and 3 (linear reaction along cellular membranes with some adjacent cytoplasmic staining in the immediate submembranous cellular zone). To detect collagen, we used rabbit polyclonal anti-collagen I and mouse monoclonal anti-collagen IV antibodies (Abcam, Cambridge, United Kingdom, both). In the case of collagen I, the staining was scored as 0 (lack of staining), 1 (very faint staining), 2 (evident positive stain), 3 (strong reaction), and the percentage of stroma showing reactivity was estimated. In collagen IV, the staining was scored 0–3, as described for collagen I. Representative pictures of immunohistochemistry scoring are provided in Supplementary Figure S3.

IHC staining was obtained from all subjects studied, whereas reliable RBM measurements from 36 patients with persistent airflow limitation, 35 with non-persistent airflow limitation, and 24 controls. 

### 4.6. Lung Computed Tomography and Airway Cross-Sectional Geometry

Lung CT was performed 1 h after administration of 400 μg of albuterol using 64-raw multidetector computed tomograph (Aquilion TSX-101A, Toshiba Medical Systems Corporation, Otawara, Japan) in a helical scanning mode (CT parameters: 64 × 0.5 mm collimation, the helical pitch of 53 and 0.5 s per rotation with standard radiation dose [150 ± 50 mAs and 120 kVp]) and without administration of contrast medium. Automated program AW Server (General Electric Healthcare, Wauwatosa, WI, USA) was used to quantify the airway cross-sectional geometry in four different bronchi: the right upper lobe apical segmental bronchus (RB1), the right lower lobe basal posterior bronchus (RB10), the left apicoposterior bronchus (LB1 + 2), and the left lower posterior basal bronchus (LB10). Patients were scanned in the supine position at maximal inspiration with their arms held over their heads. After identifying the studied bronchus, the operator placed a seed point in the airway lumen. The software automatically quantified airway geometry parameters, including average diameter of the lumen and airway, average wall thickness, lumen and wall area, wall area ratio (WAR), and wall thickness ratio (WTR) [27,39]. The wall thickness was calculated based on the difference between the average outer and inner bronchial diameters. The WAR was defined as a difference between the average outer and inner area divided by the average outer area. The WTR was calculated as the ratio of the average wall thickness and the airway diameter. CT-based indices were measured blindly two times by an independent radiologist (J.Z.) on different days; subsequently, the mean value was calculated. 

Among the initially enrolled subjects, two asthmatics were excluded due to lobar atelectasis, four with a lung tumor, three with enlarged lymph nodes of the mediastinum, and three with bronchiectasis. Moreover, six asthma patients had emphysema, whereas, in the following ten and two controls, reliable automatic measurements of airway remodeling indices were not possible. Moreover, two control subjects were excluded due to abnormal BAL cellularity, i.e., 4% of eosinophils in one case and 40% of lymphocytes in the second one. Finally, we enrolled 47 asthma subjects with persistent airflow limitation, 45 with non-persistent airway obstruction, and 36 control individuals for further analysis.

### 4.7. Statistical Analysis 

Data were compared by STATISTICA 13.5 software. The Shapiro–Wilk test was used to verify data distribution. Continuous variables, mostly non-normally distributed, were reported as medians with interquartile range or means with standard deviation and compared by the Mann–Whitney U-test or unpaired t-test, as appropriate. Categorical variables were given as percentages and compared by χ^2^ test. Age, sex, and BMI were considered potential confounders. Therefore, the main data were Box–Cox transformed (or entered in original scale if normally distributed), and one-way covariance analysis (ANCOVA) was performed to adjust for potential confounders. If appropriate, the Spearman rank correlation test or the univariate linear regression model adjusted for potential confounders were used to test associations between two continuous variables. Results were considered significant when the *p*-value was less than 0.05.

## 5. Conclusions

Our data suggest that separate mechanisms may operate in airway remodeling pathology, depending on whether they act on the airway wall structure (e.g., smooth muscle hypertrophy) or at the site of bronchial mucosa (e.g., RBM thickness and collagen deposition).

Additionally, the results presented here imply that extracellular matrix components, e.g., collagen I, likely interact via α_2_β_1_ integrin on airway epithelium and infiltrating inflammatory cells, inhibiting the advancement of bronchial remodeling, including RBM thickening and collagen I accumulation. However, largely observational and experimental studies are needed to explain the mechanisms and clinical relevance of that interplay in asthma severity, progression, and potential therapeutic modality.

## Figures and Tables

**Table 1:** Demographic and clinical characteristics of the subjects studied.

	Non-Persistent Airflow Limitation *n* = 45	Persistent Airflow Limitation *n* = 47	Control *n* = 36	*p*-Value Non-Persistent vs. Persistent Limitation	*p*-Value Non-Persistent Limitation vs. Control	*p*-Value Persistent Limitation vs. Control
**Demographic variables**						
Age, years	52 (41–59)	58 (52–65)	55 (45–65)	**0.004**	0.07	0.27
Male gender, *n* (%)	10 (22)	16 (34)	5 (14)	0.15	0.5	0.07
Body mass index, kg/m^2^	27.8 (24.8–30.8)	26.4 (23.5–31.6)	27.3 (23.0–27.9)	0.53	0.13	0.95
**Smoking history**						
Past smoking, *n* (%)	13 (29)	15 (32)	12 (33)	0.93	0.85	0.92
Pack-years of smoking	0 (0–7)	0 (0–8)	0 (0–4)	0.85	0.84	0.9
**Comorbidities**						
Atopy, *n* (%)	27 (60)	23 (49)	6 (17)	0.39	**0.0002**	**0.005**
GERD, *n* (%)	16 (36)	22 (47)	23 (64)	0.38	**0.02**	0.19
Arterial hypertension, *n* (%)	18 (40)	28 (60)	15 (42)	0.09	0.94	0.16
Diabetes mellitus, *n* (%)	6 (13)	12 (26)	3 (8)	0.23	0.72	0.08
Hypercholesterolemia, *n* (%)	9 (20)	16 (34)	6 (17)	0.2	0.92	0.13
Coronary heart disease, *n* (%)	2 (4)	5 (11)	2 (6)	0.47	0.77	0.67
**Asthma-related variables**						
Asthma duration, years	11.5 (5–19.5)	10 (7–20)		0.86		
Asthma severity (GINA): persistent mild, *n* (%) persistent moderate, *n* (%) persistent severe, *n* (%)	8 (18) 22 (49) 15 (33)	7 (15) 15 (32) 25 (53)		0.14		
Asthma treatment: Inhaled corticosteroids, *n* (%) Long-acting β2-agonists, *n* (%) Montelukast, *n* (%) Theophylline, *n* (%) Oral corticosteroids, *n* (%)	45 (100) 31 (69) 9 (20) 4 (9) 8 (18)	47 (100) 42 (89) 4 (9) 10 (21) 15 (32)		0.17		
**Spirometry results**						
FEV_1_ before bronchodilator, L	2.79 ± 0.76	1.79 ± 0.8	2.71 ± 0.75	**<0.001**	0.65	**<0.001**
FEV_1_ before bronchodilator, % of the predicted value	100.3 (89.5–111.1)	66.7 (54.1–80.6)	110.9 (106.8–114.7)	**<0.00**1	**<0.001**	**<0.001**
FEV_1_ after bronchodilator, L	2.92 ± 0.73	2.07 ± 0.95	2.84 ± 0.79	**<0.001**	0.66	**<0.001**
FEV_1_ after bronchodilator, % of the predicted value	103.8 (96.4–116.5)	79.2 (62.8–87.2)	116 (112.1–122.3)	**<0.001**	**<0.001**	**<0.001**
FEV_1_/VC (before bronchodilator)	73.3 (67.8–78.18)	59.1 (51.7–63.8)	74.84 (73.23–78.38)	**<0.001**	0.16	**<0.001**
FEV_1_/VC (after bronchodilator)	76.99 (73.05–81.88)	65.4 (54.5–68.6)	79.33 (77.25–80.38)	**<0.001**	0.25	**<0.001**

Table 1 footnote. Categorical variables are presented as numbers (percentages), continuous variables as median and interquartile range, or mean and standard deviation, as appropriate. Abbreviations: GERD—Gastroesophageal reflux disease, GINA—Global Initiative for Asthma, FEV_1_—Forced expiratory volume in 1 s, VC—Vital capacity, L-liter, *n*—number. Statistics: Mann–Whitney U-test or unpaired t-test, as appropriate.

**Table 2:** Laboratory variables.

	Reference Range	Non-Persistent Airflow Limitation *n* = 45	Persistent Airflow Limitation *n* = 47	Control *n* = 36	*p*-Value Non-Persistent vs. Persistent Limitation	*p*-Value Non-Persistent Limitation vs. Control	*p*-Value Persistent Limitation vs. Control
**Basic laboratory tests**							
Red blood cells, 10^6^/μL	4–5	4.65 ± 0.4	4.7 ± 0.5	4.48 ± 0.4	0.68	**0.048**	**0.03**
White blood cells, 10^3^/μL	4–10	6.26 (5.43–7.33)	7.44 (6.39–9.25)	5.44 (5.16–7.08)	**<0.001**	0.07	**<0.001**
Neutrophils, 10^3^/μL	1.8–7.7	3.1 (2.7–4.1)	3.7 (2.9–4.8)	3.3 (2.9–3.6)	**0.049**	0.76	**0.04**
Lymphocytes, 10^3^/μL	1–4.5	1.94 (1.58–2.43)	2.2 (1.58–2.61)	1.65 (1.44–2.08)	0.5	**0.03**	**0.03**
Monocytes, 10^3^/μL	0.1–0.8	0.57 (0.49–0.74)	0.71 (0.53–0.9)	0.49 (0.41–0.62)	**0.02**	**0.006**	**<0.00**1
Blood platelets, 10^3^/μL	140–400	223 (193–247)	225 (191–265)	228 (189–246)	0.78	0.98	0.85
**Asthma and inflammatory biomarkers (blood)**							
Eosinophilia/μL	40–450	230 (130–310)	400 (180–680)	110 (70–170)	**0.009**	**<0.001**	**<0.001**
Immunoglobulin E, IU/mL	0–100	90 (26–400)	88 (43–511)	23 (18–48)	0.6	**<0.00**1	**<0.001**
C-reactive protein, mg/L	0–5	1.64 (0.53–8)	4.53 (0.58–9.38)	1.78 (0.89–2.29)	0.39	0.28	**0.008**
Fibrinogen, g/L	1.8–3.5	3.1 (2.8–3.5)	3.5 (3.2–4.2)	2.9 (2.3–3.7)	**0.03**	0.11	**0.002**
Periostin, ng/mL	0.29–0.61 ^§^	0.28 (0.24–0.33)	0.38 (0.31–0.51)	0.37 (0.36–0.45)	**0.01**	**0.001**	0.85
Interleukin 6, pg/mL	0.005–1.432 ^§^	0.72 (0.43–1.19)	1.09 (0.47–2.38)	0.57 (0.43–0.97)	0.14	0.29	**0.03**
Interleukin 10, pg/mL	0.163–1.022 ^§^	0.6 (0.22–1.06)	0.55 (0.35–0.89)	0.43 (0.2–0.76)	0.95	0.17	0.1
Interleukin 12 (p70), pg/mL	0.005–2.618 ^§^	0.005 (0.005–1.2)	0.005 (0.005–1.25)	0.005 (0.005–0.33)	0.7	0.13	0.26
ADAM-33, ng/mL	0.083–2.257 ^§^	0.73 (0.2–1.29)	1.32 (0.33–2.37)	0.41 (0.13–1.5)	**0.01**	0.65	**0.007**
**Circulating integrin subunits**							
α_1_ integrin, ng/mL	6.45–103.67 ^§^	17.32 (6.88–52.4)	32.7 (14.7–55.7)	24.1 (8.90–76.5)	0.14	0.21	0.83
α_2_ integrin, ng/mL	7.79–36.19 ^§^	15.5 (9.7–25.5)	22.9 (15–39)	20.5 (11.7–26.5)	**0.03**	0.25	0.21
**Asthma and inflammatory biomarkers (bronchoalveolar lavage fluid)**							
Periostin, ng/mL	0.1–1.15 ^§^	0.86 (0.8–0.99)	0.81 (0.72–0.95)	0.8 (0.51–0.88)	0.34	0.17	0.49
Eosinophils, %	0–1 ^#^	0.5 (0–1)	1 (0.1–3)	0.1 (0–1)	**0.02**	0.62	**0.006**

Table 2 footnote. Variables are presented as median and interquartile range or mean and standard deviation, as appropriate; ^§^—reference range based on the 10th and 90th percentile values in the control group, ^#^—reference according to the American Thoracic Society Guidelines [26]; Abbreviation: ADAM-33—a disintegrin and metalloproteinase domain-containing protein 33. Statistics: Mann–Whitney U-test or unpaired t-test, as appropriate.

**Table 3:** Results of histology investigations.

	Non-Persistent Airflow Limitation *n* = 45	Persistent Airflow Limitation *n* = 47	Control *n* = 36	*p*-Value Non-Persistent vs. Persistent Limitation	*p*-Value Non-Persistent Limitation vs. Control	*p*-Value Persistent Limitation vs. Control
Collagen I, % of the stroma showing reactivity in bronchial mucosa section	30 (20–60)	30 (20–60)	30 (20–70)	0.77	0.67	0.89
Collagen I, strength of the staining	1 (0–1)	1 (0–1)	2 (1–2)	0.53	0.86	0.59
Collagen IV, strength of the staining	1 (1–2)	1 (1–2)	2 (1–2)	0.99	0.27	0.3
α_1_ integrin, strength of the staining	1 (1–2)	1 (1–2)	2 (1–2)	0.31	0.13	0.61
α_2_ integrin, strength of the staining	1 (0–1)	1 (0–2)	1 (0–2)	0.66	0.24	0.12
Thickness of the reticular basement membrane of bronchial mucosa, μm	6.77 (5.85–7.81)	6.2 (5.5–7.3)	4.77 (4.3–5.41)	0.66	**0.0002**	**0.0001**

Table 3 footnote. Variables are presented as a median and interquartile range. The reticular basement membrane thickness was measured in *n* = 35 (78%) asthma patients with non-persistent airflow limitation, *n* = 36 (77%) of those with persistent airway obstruction, and *n* = 24 (67%) control. Statistics: continuous variables: one-way covariance analysis (ANCOVA) adjusted for age, sex, and body mass index; categorical variables: χ^2^ test.

**Table 4:** Indices of lung computed tomography airway remodeling.

	Non-Persistent Airflow Limitation *n* = 45	Persistent Airflow Limitation *n* = 47	Control *n* = 36	*p* *-Value Non-Persistent vs. Persistent Limitation	*p* *-Value Non-Persistent Limitation vs. Control	*p* *-Value Persistent Limitation vs. Control
Total lung volume, L	5.07 ± 1.05	5.49 ± 1.4	4.74 ± 1.09	0.3	0.18	0.07
Low-attenuation lung area (threshold level of −950 Hounsfield units), %	7.53 (2.3–18.44)	18.26 (4.1–30.56)	11.14 (2.3–23.28)	0.26	0.85	0.48
**The right upper lobe apical segmental bronchus (RB1)**						
Lumen diameter, mm	4.24 ± 0.95	4.12 ± 1.06	4.29 ± 0.85	0.07	0.73	0.07
Airway diameter, mm	8.02 ± 1.47	8.18 ± 1.9	7.98 ± 1.45	0.34	0.46	0.54
Wall thickness, mm	1.8 (1.6–2.1)	1.9 (1.7–2.3)	1.8 (1.7–2.1)	0.74	0.69	0.6
Wall thickness ratio (WTR)	23.5 (21.7–25)	24.8(22.9–26.9)	23.6 (21.8–24.2)	**0.03**	0.94	**0.009**
Lumen area, mm^2^	12.2 (10–17.4)	12.5 (9.5–16)	14.5 (9.1–16.3)	0.06	0.22	0.32
Wall area, mm^2^	34.9 (27.2–48.3)	35 (27.4–45.4)	33 (28.6–43.4)	0.63	0.49	0.86
Wall area ratio (WAR)	72 (68.3–75.3)	75 (71.3–78.7)	72.2 (68.5–74)	**0.02**	0.98	**0.006**
**The right lower lobe basal posterior bronchus (RB10)**						
Lumen diameter, mm	4.3 (3.6–4.9)	3.7 (3.3–4.5)	4.4 (4.3–4.8)	**0.003**	0.67	**0.001**
Airway diameter, mm	7.9 (6.8–9.3)	7.7 (6.6–8.5)	8 (7.4–8.5)	**0.02**	0.63	0.22
Wall thickness, mm	1.8 (1.6–1.9)	1.8 (1.6–2.1)	1.7 (1.5–1.8)	0.73	0.25	0.18
Wall thickness ratio (WTR)	23.3 (20.1–24.5)	24.1 (22.9–26.4)	21.9 (20–24.5)	**0.009**	0.17	**0.001**
Lumen area, mm^2^	13.9 (8.9–18.9)	10.8 (8.7–15.8)	14.9 (14.2–18)	**0.005**	0.53	**0.002**
Wall area, mm^2^	34.4 (27–45.8)	33.7 (24.9–42)	32.3 (21.4–36.3)	0.09	0.31	0.8
Wall area ratio (WAR)	71.8 (64.1–73.9)	73.4 (70.7–77.9)	68.9 (63.9–73.9)	**0.008**	0.18	**0.0009**
**The left apicoposterior bronchus (LB1 + 2)**						
Lumen diameter, mm	5.09 ± 1.09	5.04 ± 1.15	5 ± 1.15	0.79	0.69	0.86
Airway diameter, mm	9.3 (8–10.3)	9.8 (8.3–10.4)	8.6 (7.4–10.7)	0.81	0.52	0.53
Wall thickness, mm	2 (1.7–2.2)	2.2 (1.9–2.4)	1.8 (1.6–2.3)	0.09	0.16	0.03
Wall thickness ratio (WTR)	22.4 ± 2.7	23.5 ± 2.9	22.8 ± 3.7	0.17	0.61	0.67
Lumen area, mm^2^	20.2 (13.4–28.3)	22.1 (14.2–27.8)	15.2 (13.2–26.3)	0.76	0.69	0.88
Wall area, mm^2^	46.6 ± 13.7	50.9 ± 16.2	45.7 ± 15	0.42	0.77	0.36
Wall area ratio (WAR)	69.3 ± 6	71.5 ± 6.2	69.7 ± 8.1	0.18	0.7	0.62
**The left lower posterior basal bronchus (LB10)**						
Lumen diameter, mm	4.3 (3.9–4.7)	4.2 (3.5–5)	5.1 (4–5.6)	**0.04**	0.41	**0.0003**
Airway diameter, mm	8.33 ± 1.3	8.1 ± 1.4	8.9 ± 1.7	0.13	0.13	**0.003**
Wall thickness, mm	1.9 (1.7–2.1)	1.8 (1.7–2.2)	2 (1.7–2.1)	0.55	0.69	0.44
Wall thickness ratio (WTR)	23.2 (21.8–25)	24.2 (21.8–26.1)	22.4 (21.7–24.3)	0.16	0.15	**0.009**
Lumen area, mm^2^	14.3 (11.8–17.2)	13.8 (9.9–19.5)	19.8 (10.4–24.8)	0.14	0.25	**0.01**
Wall area, mm^2^	36.4 (31.1–46.6)	36.7 (29.6–45.9)	43.3 (28.2–52.8)	0.24	0.48	0.13
Wall area ratio (WAR)	71.3 (68.3–75.3)	73.6 (68.1–77.2)	69.3 (65.3–73.7)	0.06	0.07	**0.001**

Table 4 footnote. Variables are presented as a median and interquartile range, or mean and standard deviation, as appropriate. Statistics: one-way covariance analysis (ANCOVA) adjusted for age, sex, and body mass index; *—*p*-value adjusted for age, sex, and body mass index.

## Data Availability

The data presented in this study are available on request from the corresponding author. The data are not publicly available due to patients’ origin.

## Supplementary Materials

This article involves an online Supplement. The following are available online at https://www.mdpi.com/article/10.3390/ijms22126315/s1, Table S1: Additional laboratory variables. Table S2: Differences in the percentage of T-cells expressing studied integrin chains in comparison of asthma patients with persistent or non-persistent airflow limitation and control subjects. Table S3: Differences in the percentage of eosinophils expressing studied integrin chains in comparison of asthma patients with persistent or non-persistent airflow limitation and control subjects. Figure S1: Design of flow cytometry panels and gating strategy used to measure the surface integrin expression in blood T-cells and eosinophils. Figure S2: Design of flow cytometry panels and gating strategy used to measure the surface expression of integrin chains on bronchoalveolar lavage (BAL) T-cells and eosinophils. Figure S3: Representative immunohistochemistry images (600×) of α_1_ integrin subunit, α_2_ integrin subunit, type I collagen, and type IV collagen expression in the bronchial mucosa.
