# Supplementary material for: Interactions Via α2β1 Cell Integrin May Protect against the Progression of Airway Structural Changes in Asthma
Source: Int J Mol Sci. 2021 Jun 12;22(12):6315. doi: 10.3390/ijms22126315 (PMC8231566; doi:10.3390/ijms22126315)
Supplement: Supplementary file 1 [file ijms-22-06315-s001.zip › ijms-1237766-supplementary.pdf]

Supplementary Data

Interactions via $\alpha_2\beta_1$ cell integrin may protect against the progression of airway structural changes in asthma

Stanislawa Bazan-Socha, Bogdan Jakiela, Joanna Zuk, Jacek Zarychta, Jerzy Soja, Krzysztof Okon, Sylwia Dziedzina, Lech Zareba, Jerzy Dropinski, Krzysztof Wojcik, Agnieszka Padjas, Cezary Marcinkiewicz, Jan G. Bazan

Correspondence to:

Stanislawa Bazan-Socha, MD, PhD

Department of Internal Medicine, Jagiellonian University Medical College

8 Skawinska Str

31-066 Krakow, Poland

Tel: +48-12-4305266

Fax: +48-12-4305203

E-mail: mmsocha@cyf-kr.edu.pl

Supplementary Text

Methods

Characteristics of the patients

"Mild" asthma was defined as a mild persistent disease, treated with a low daily dose of inhaled corticosteroids (ICS) (<250 μg of fluticasone propionate [FP] [dry powder inhaler] or equivalent). "Moderate" asthma was defined as a mild persistent disease treated with low (combined with long-acting β2-agonists) or medium dose of ICS (250-500 μg of FP or equivalent). "Severe" asthma was defined as a disease requiring a high ICS dose (>500 μg of FP or equivalent) with a long-acting β2-agonist to prevent it from becoming uncontrolled or asthma that remains uncontrolled despite this treatment [1].
The exclusion criteria included pregnancy or breastfeeding, any acute illness, congestive heart failure, atrial fibrillation, myocardial infarction or stroke, cancer, hyper- or hypothyroidism, liver injury, and chronic kidney disease (stage 3 or more) in a history. Arterial hypertension, diabetes mellitus, hypercholesterolemia, or stable coronary heart disease were allowed in study participants.
Diabetes mellitus (DM) was defined as the current use of insulin or oral hypoglycemic medications or fasting serum glucose >7.0 mmol/l. Arterial hypertension was defined based on a history of hypertension (blood pressure >140/90 mmHg) or administration of antihypertensive treatment. Hypercholesterolemia was defined as previously diagnosed and treated with statins or serum total cholesterol >5.2 mmol/l. Liver injury was diagnosed if serum alanine aminotransferase was elevated (at least two times above the upper limit of the reference range). Coronary heart disease was defined as a documented history of coronary stenosis or stable angina.

Supplementary Tables

Table S1. Additional laboratory variables.

	**Reference range**	**Non-persistent airflow limitation n=45**	**Persistent airflow limitation n=47**	**Control n=36**	**p - value non-persistent vs. persistent limitation**	**p-value non-persistent limitation vs. control**	**p-value persistent limitation vs. control**
**Asthma and inflammatory biomarkers (blood)**							
Interleukin 4, pg/ml	0.005-0.005§	0.005 (0.005-0.005)	0.005 (0.005-0.005)	0.005 (0.005-0.005)	0.99	0.83	0.82
Interleukin 5, pg/ml	0.005-0.005§	0.005 (0.005-0.005)	0.005 (0.005-0.005)	0.005 (0.005-0.005)	0.99	0.43	0.42
Interleukin 17A, pg/ml	0.005-0.665§	0.005 (0.005-0.2)	0.005 (0.005-0.12)	0.005 (0.005-0.17)	0.87	0.72	0.87
Interleukin 23, pg/ml	0.005-78.6§	0.005 (0.005-17.1)	0.005 (0.005-21.2)	0.005 (0.005-33.2)	0.59	0.53	0.3
Interferon γ, pg/ml	0.005-0.528§	0.04 (0.005-0.46)	0.005 (0.005-0.05)	0.038 (0.005-0.41)	**0.03**	0.64	**0.04**
**Asthma and inflammatory biomarkers (bronchoalveolar lavage fluid)**							
Interleukin 4, pg/ml	0.005-4.573§	0.005 (0.005-0.16)	0.005 (0.005-0.27)	0.005 (0.005-0.57)	0.99	0.41	0.34
Interleukin 5, pg/ml	0.005-0.005§	0.005 (0.005-0.16)	0.005 (0.005-0.005)	0.005 (0.005-0.005)	0.86	0.99	0.87
Interleukin 6, pg/ml	0.005-1.547§	0.78 (0.35-1.12)	0.52 (0.005-1.41)	0.46 (0.24-1.21)	0.16	0.38	0.6
Interleukin 10, pg/ml	0.005-0.005§	0.005 (0.005-0.005)	0.005(0.005-0.005)	0.005 (0.005-0.005)	0.7	0.49	0.73
Interleukin 12 (p70), pg/ml	0.047-0.29§	0.09 (0.06-0.12)	0.07 (0.05-0.1)	0.08 (0.06-0.14)	0.22	0.82	0.2
Interleukin 17A, pg/ml	0.005-0.137§	0.01 (0.005-0.01)	0.005 (0.005-0.005)	0.005 (0.005-0.03)	0.82	0.05	0.11
Interleukin 23, pg/ml	0.005-0.005§	0.01 (0.005-0.005)	0.005 (0.005-0.005)	0.005 (0.005-0.005)	0.69	0.94	0.77
Interferon γ, pg/ml	0.005-0.005§	0.01 (0.005-0.005)	0.005 (0.005-0.005)	0.005 (0.005-0.005)	0.86	0.89	0.86
**Bronchoalveolar lavage cellularity**							
Macrophages, %	85-100#	88 (74.5-93)	84 (57-90)	87 (78-91)	0.06	0.63	0.19
Lymphocytes, %	10-15#	8 (4.5-19)	7.5 (4-14)	8 (5.5-12)	0.62	0.84	0.87
Neutrophils, %	0-3#	3 (2-4)	4 (2-11)	2 (2-6)	0.07	0.93	0.17

Table S1 footnote. Variables are presented as median and 0.25-0.75 quartiles. § - reference range based on the 10th and 90th percentile values in the control group, # - refference provided according to the American Thoracic Society Guidelines [2]; Statistics: Mann-Whitney U-test

Table S2. Differences in the percentage of T-cells expressing studied integrin chains in comparison of asthma patients with persistent or non-persistent airflow limitation and control subjects.

Integrin subunit	Para-meter	Control (n=21)	Asthma (n=87)	Non-persistent airflow limitation (n=42)	Persistent airflow limitation (n=45)
**CD4+ T-cells (blood)**					
α_1 (CD49a)	% pos.	10.2 [6.4-14.9]	11.5 [5.5-15.7]	12.6 [5.4-16.5]	10.6 [6.2-15.2]
α_2 (CD49b)	% pos.	24.1 [20-35]	23.6 [20.6-27.5]	22.3 [19.8-28.4]	24.6 [21-27.3]
α_4 (CD49d)	% pos.	77.6 [63-83.9]	78.7 [70.5-84.4]	77.9 [69.7-84.7]	78.8 [72-83.1]
α_5 (CD49e)	% pos.	74.1 [64.1-84.1]	79.1 [69.8-85.3]	79.7 [71.6-85.9]*	77.8 [67.2-85]
β_1 (CD29)†	% pos.	65.3 [57.6-71.1]	66.8 [60.8-75.2]	67.9 [60.4-78.9]	66.7 [60.8-72.5]
**CD4+ T-cells (BAL)§**					
α_1 (CD49a)	% pos.	37.7 [24.7-46.3]	49.3 [36.8-58.1]**	42.6 [34.9-54.9]	52.8 [43.6-64.5]**
α_2 (CD49b)	% pos.	40.4 [18.8-64.5]	37.6 [18.2-62]	37.3 [20.4-46.1]	38.4 [11.4-64.7]
α_4 (CD49d)	% pos.	92.2 [80.4-94.8]	91 [85.2-96.4]	93 [88.1-96.1]##	87.7 [77.2-96.5]*##
α_5 (CD49e)	% pos.	64.9 [57.6-72.5]	65.9 [54.4-80.4]	71.3 [56.3-82.2]	60.6 [52.7-77]
β_1 (CD29)†	% pos.	89.3 [85.1-96.2]	92.8 [84-97.8]	94.7 [85.4-98.2]#	89.7 [83.5-96.4]#
**CD8+ T-cells (blood)**					
α_1 (CD49a)	% pos.	6.7 [4.8-8.9]	10.1 [6.1-14.1]*	10.7 [5-16]**#	9 [6.2-13.4]#
α_2 (CD49b)	% pos.	18.9 [14.4-31.2]	18.6 [15.4-23.7]	18.9 [15.5-27.3]	18.4 [15.2-22.7]
α_4 (CD49d)	% pos.	94.6 [90-97.4]	94.6 [92.8-96.9]	94 [92-97]	95.1 [93.5-96.8]
α_5 (CD49e)	% pos.	67.7 [51.6-74.7]	70.2 [54.6-77.7]	69.5 [51.9-77.5]	71.3 [56.1-78]
β_1 (CD29)†	% pos.	75.4 [64.8-87.1]	74.7 [68.2-85.1]	73.2 [68.1-81.8]	76.5 [69.3-87.4]
**CD8+ T-cells (BAL)§**					
α_1 (CD49a)	% pos.	78.3 [67.1-91.8]	83.9 [71.3-90.3]	85.4 [70.6-92]	81.7 [75.5-89.1]
α_2 (CD49b)	% pos.	9.1 [5.4-24.2]	15.1 [9.6-23]	14.2 [9.4-20.6]	16.9 [9.2-25.9]
α_4 (CD49d)	% pos.	89 [77.4-93.7]	89.6 [82.2-95]	91.6 [85.1-95.9]##	84.6 [76.4-93.8] ##
α_5 (CD49e)	% pos.	44.2 [30.4-51.7]	34.2 [24.3-50.3]	37.1 [22.7-55.2]	33.3 [25-46.9]
β_1 (CD29)†	% pos.	82.9 [77.8-89.9]	89.9 [84-93.3]	90 [84-94.2]	88.2 [83.7-92.7]

Table S3 footnote.

Data presented as medians and 0.25-0.75 quartiles. ANCOVA with adjustment for age, sex, and BMI statistics: *P<0.05, **P<0.01 in comparison to control group; #P<0.05, ##P<0.01 persistent vs. non-persistent airflow limitation group. § - Due to technical reasons, integrin expression on BAL lymphocytes was measured in 93% of samples, i.e., control (n=21), non-persistent airflow limitation (n=40), persistent airflow limitation (n=40). † - Expression of β_1 (CD29) was analyzed in 79% of samples.

Table S3. Differences in the percentage of eosinophils expressing studied integrin chains in comparison of asthma patients with persistent or non-persistent airflow limitation and control subjects.

**Integrin subunit**	**Para-meter**	**Control** (n=19)	**Asthma** (n=84)	**Non-persistent airflow limitation** (n=41)	**Persistent airflow limitation** (n=43)
**Eosinophils (blood)**					
α_1 (CD49a)	% pos.	2.2 [0.7-5.8]	5.8 [1.8-14.6]*	8.4 [2.2-14.5]*	5.4 [1.3-15.3]*
α_2 (CD49b)	% pos.	13.4 [8.9-16.2]	11.6 [9.4-17.4]	13.9 [10.4-18.6]	11.1 [6.2-15]
α_4 (CD49d)	% pos.	99.9 [99.8-100]	99.9 [99.8-100]	99.9 [99.8-100]	99.9 [99.9-100]
α_5 (CD49e)	% pos.	2 [0.9-3.7]	1.6 [0.8-3.5]	1.6 [0.8-4.5]	1.6 [0.8-3.1]
β_1 (CD29)†	% pos.	79 [59.9-88.9]	62.5 [48.4-89.5]	73.3 [54.4-95.5]	56 [45.6-83.7]
**Eosinophils (BAL)§**					
α_1 (CD49b)	% pos.	11.2 [3.9-21.4]	4.9 [2.4-11.9]	4.6 [1.4-11.1]	4.9 [3.7-13]
α_2 (CD49b)	% pos.	3.5 [1.1-6.3]	2.5 [1.7-4]	2.2 [1-3]	3 [2.3-4.3]
α_4 (CD49d)	% pos.	64.8 [49.7-74.7]	60.8 [43.6-81.6]	62.2 [35.2-82.5]	60.8 [50-81.6]
α_5 (CD49e)	% pos.	7.7 [5.3-34.3]	4.4 [2.5-10]*	5.9 [3.3-11.1]	3.7 [2-6.6]
β_1 (CD29)†	% pos.	8.4 [4.6-13.5]	7.3 [3.9-10.1]	7.9 [5.3-12.2]	5.8 [2.7-8.4]

Table S4 footnote.

Data presented as medians and 0.25-0.75 quartiles. ANCOVA with adjustment for age, sex, and BMI statistics: *$P<0.05$, in comparison to control group. § - Due to the variable degree of airway eosinophilia, only 34% of samples were analyzed for integrin expression on BAL eosinophils, i.e., control (n=4; reliable comparison to other groups is not possible), non-persistent airflow limitation (n=16), persistent airflow limitation (n=15). † - Expression of β_1 (CD29) was analyzed in 78% of samples.

Supplementary Figures

Supplementary Figure S1

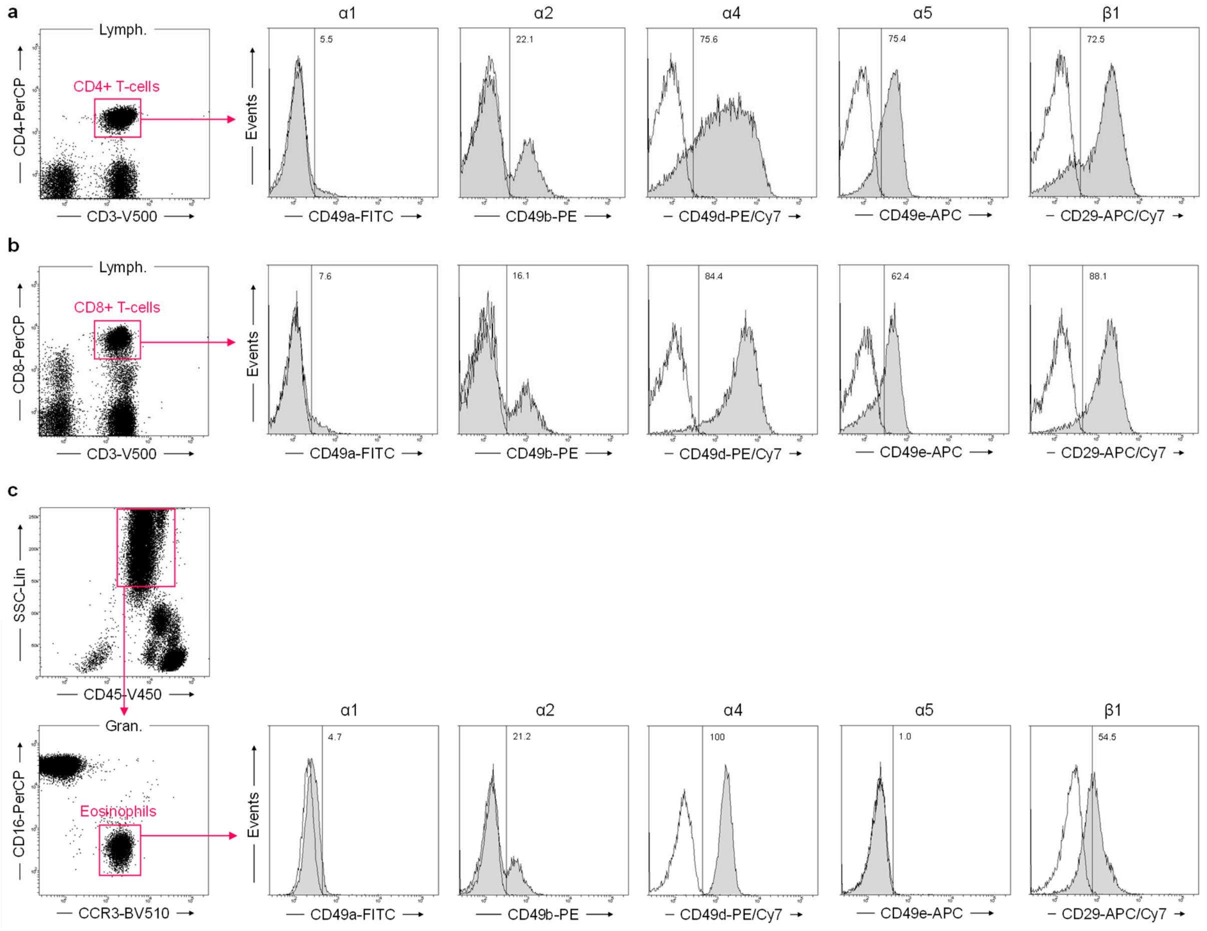

Figure S1 legend. Design of flow cytometry panels and gating strategy used to measure the surface integrin expression in blood T-cells and eosinophils. Aliquots of EDTA anticoagulated blood were stained using 3 sets of premixed antibodies (7 or 8-color panels), including the five antibodies detecting integrin chains and additional 3 sets with a mixture of gating antibodies (e.g., CD3 and CD4) and all required isotype controls. Samples were analyzed on FACS Canto II cytometer with gates set on the studied cell populations and corresponding control samples. (**a**) Representative flow cytometry dot plot (CD3-V500, CD4-PerCP) with assigned CD4+ T-cell region (gated on FSC/SSC lymphocytes [Lymph.], not shown), and overlay histograms (not smoothed) showing fluorescence distribution of each analyzed integrin chain (shaded histograms; CD49a-FITC, CD49b-PE, CD49d-PE/Cy7, CD49e-APC, and CD29-APC/Cy7) compared to matched isotype controls (transparent histograms). (**b**) Flow cytometry dot plots and histograms showing expression of integrins within CD8+ (CD3-V500, CD8-PerCP) subset. (**c**) Gating strategy to identify granulocytes (Gran., FSC/SSC gate, CD45-V450 positive) and eosinophils (CCR3-BV510 positive, CD16-PerCP negative) and representative histograms showing isotype control (transparent) or specific antigen (shaded) staining of integrin subunits gated on eosinophils. Numeric values in each histogram refer to %-positive cells (markers were set on controls [<1% positive]).

Supplementary Figure S2

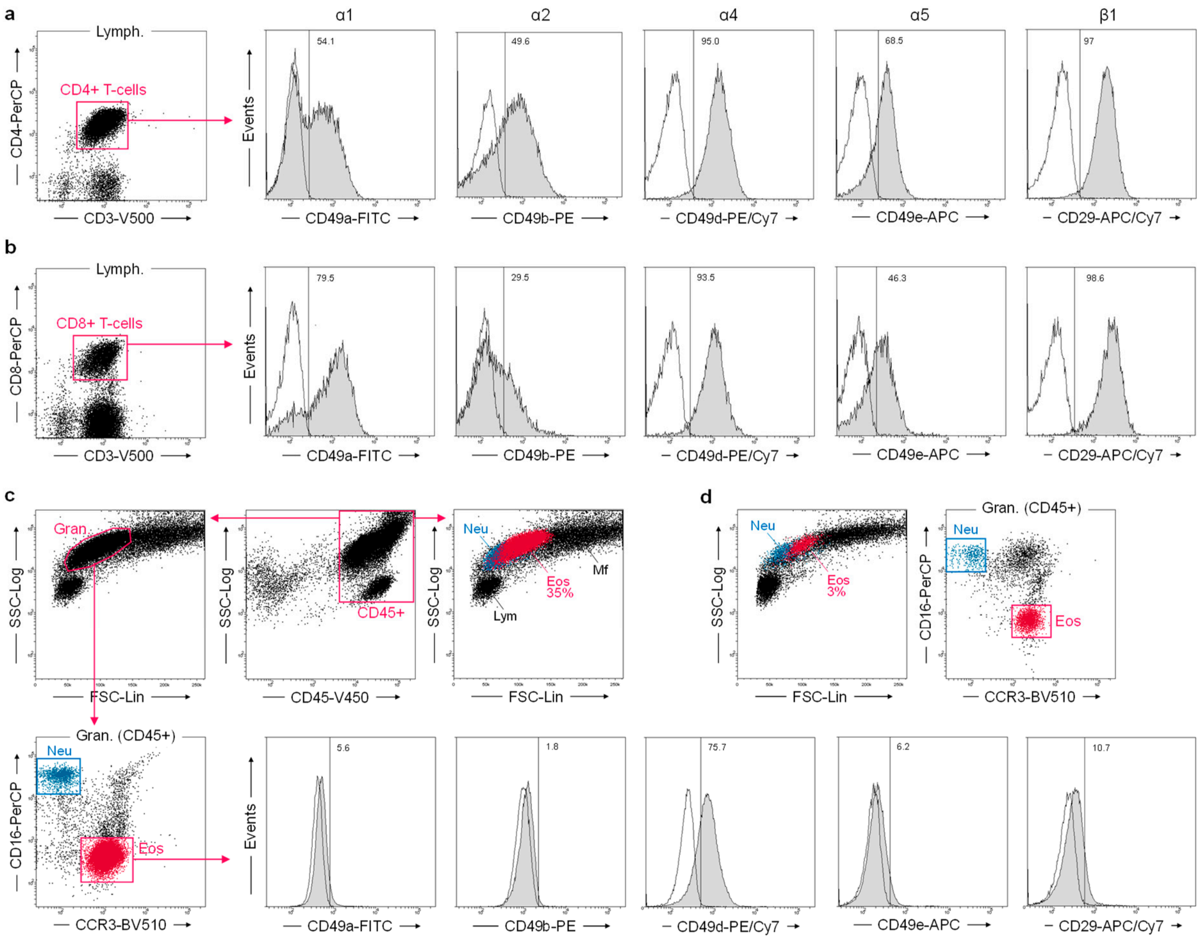

Figure S2 legend. Design of flow cytometry panels and gating strategy used to measure the surface expression of integrin chains on bronchoalveolar lavage (BAL) T-cells and eosinophils. (**a**) Representative flow cytometry dot plot (gated on lymphocytes [Lymph.] in FSC/SSC plot, not shown) with CD4+ T-cell region and overlay histograms (not smoothed) showing fluorescence distribution of analyzed integrin chains (shaded histograms; CD49a-FITC, CD49b-PE, CD49d-PE/Cy7, CD49e-APC, and CD29-APC/Cy7) compared to matched isotype controls (transparent histograms). (**b**) Flow cytometry dot plot and histograms showing expression of integrins within CD8+ (CD3-V500, CD8-PerCP) subset. (**c**) Gating strategy used to identify BAL eosinophils. Sample with 35% of eosinophils based on cytospin differential count is shown as an example. We first identified CD45+ granulocytes (Gran.) based on CD45-V450 staining and FSC/SSC plot (shown on the left). Next, gated cells were analyzed in CCR3-BV510 vs. CD16-PerCP plot (lower panel), which allowed for the identification of eosinophils (CCR3-positive, CD16-negative; *red*) and neutrophils (*blue*). These two cell populations are color-marked in the additional FSC/SSC plot (on the right; Mf – macrophages. Lym – lymphocytes, Neu - neutrophils). The lower set of plots shows overlay histograms with specific antigen staining (shaded) on BAL eosinophils and matched isotype controls (transparent). Numeric values in each histogram refer to %-positive cells (markers were set on controls [<1% positive]). (**d**) Flow cytometry dot plots (FSC/SSC gated on CD45+, and CCR3 vs. CD16 gated on granulocytes) of BAL specimen from a patient with marginally increased airway eosinophilia (3% of BAL cells) shown for comparison (gating strategy as in 'c').

Supplementary Figure S3

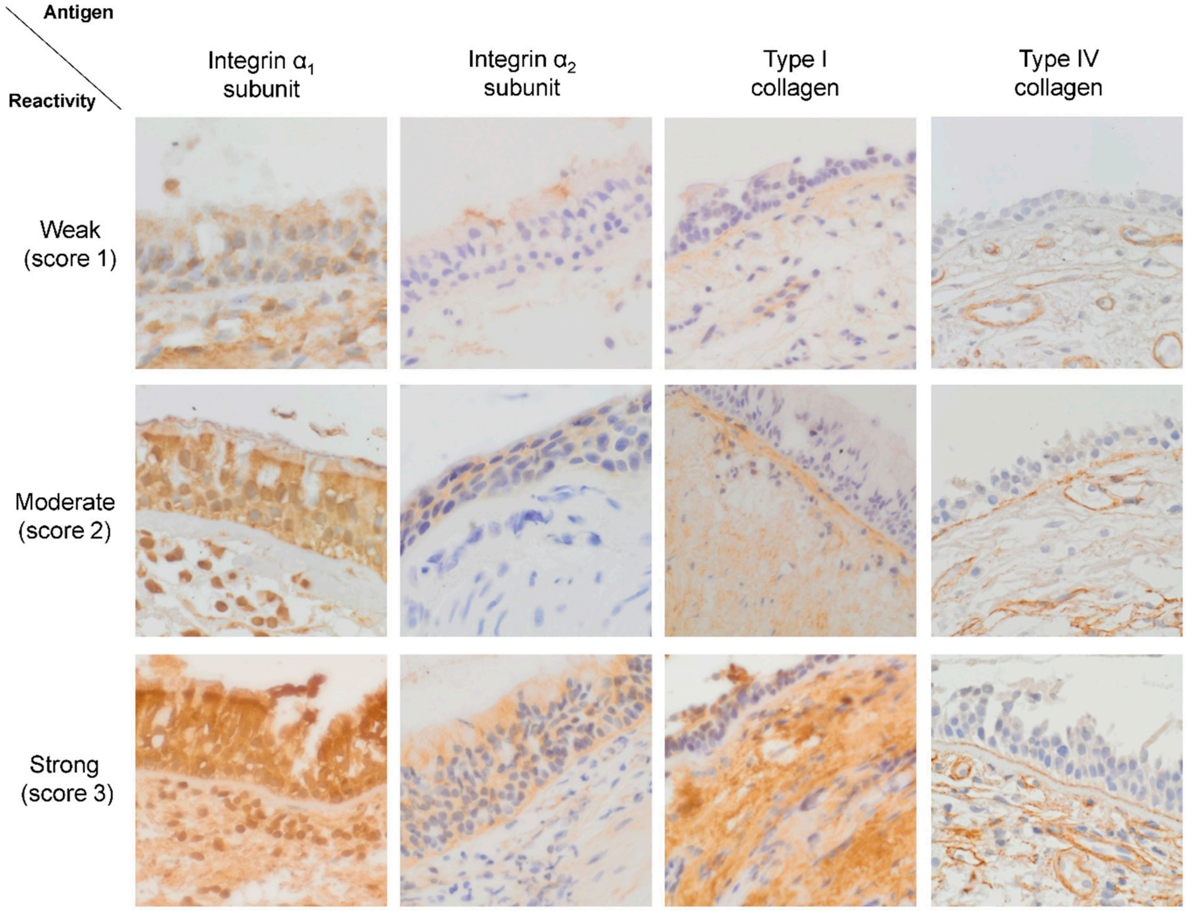

Figure S3 legend. Representative immunohistochemistry images (600x) of α_1 integrin subunit, α_2 integrin subunit, type I collagen, and type IV collagen expression in the bronchial mucosa (slides counterstained with hematoxylin and eosin) showing different staining reactivity (scoring system 1-3, score 0 [no reactivity] is not shown). Please see the additional description in the Methods section of the main article.

References

(1) Global Initiative for Asthma - Global Initiative for Asthma - GINA https://ginasthma.org/ (accessed Sep 26, 2018).

(2) Meyer, K. C.; Raghu, G.; Baughman, R. P.; Brown, K. K.; Costabel, U.; Du Bois, R. M.; Drent, M.; Haslam, P. L.; Kim, D. S.; Nagai, S.; Rottoli, P.; Saltini, C.; Selman, M.; Strange, C.; Wood, B. An Official American Thoracic Society Clinical Practice Guideline: The Clinical Utility of Bronchoalveolar Lavage Cellular Analysis in Interstitial Lung Disease. *American Journal of Respiratory and Critical Care Medicine*. Am J Respir Crit Care Med May 1, 2012, pp 1004–1014. https://doi.org/10.1164/rccm.201202-0320ST.
